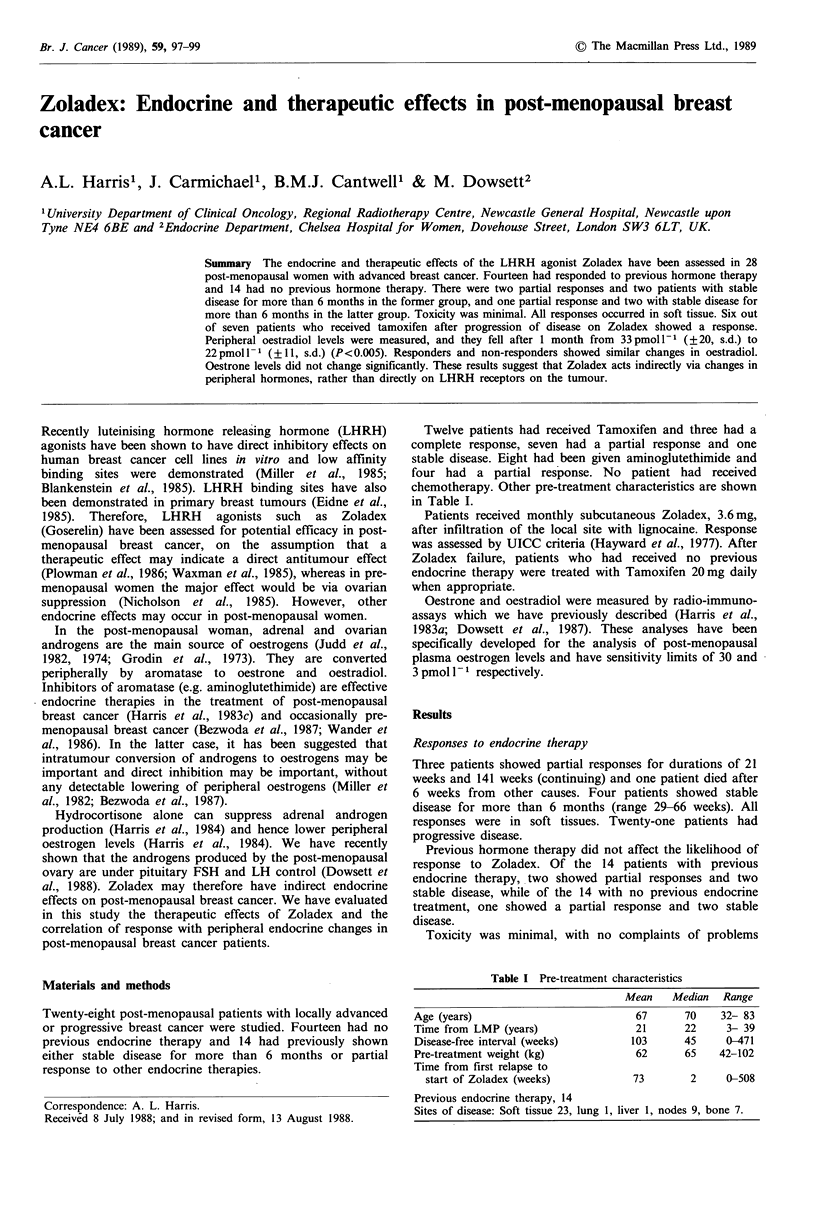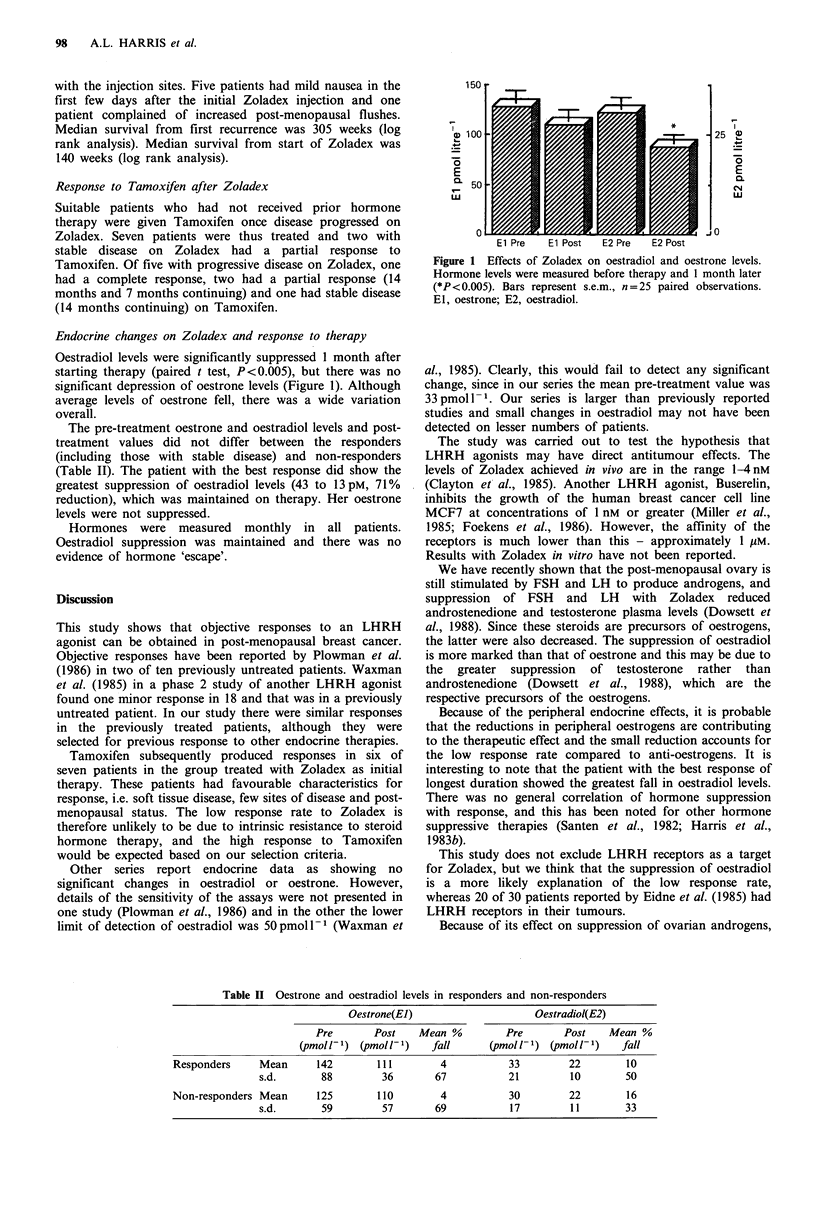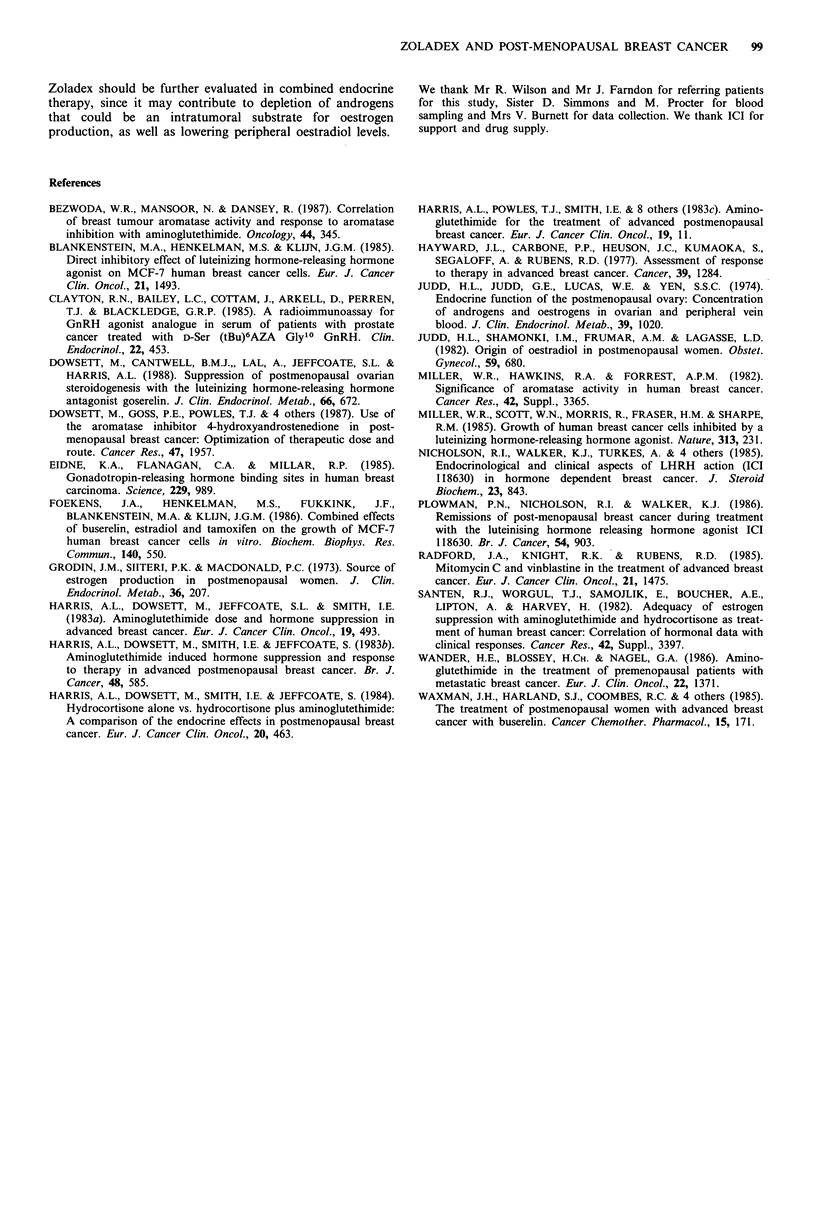# Zoladex: endocrine and therapeutic effects in post-menopausal breast cancer.

**DOI:** 10.1038/bjc.1989.19

**Published:** 1989-01

**Authors:** A. L. Harris, J. Carmichael, B. M. Cantwell, M. Dowsett

**Affiliations:** University Department of Clinical Oncology, Newcastle General Hospital, Newcastle upon Tyne.

## Abstract

The endocrine and therapeutic effects of the LHRH agonist Zoladex have been assessed in 28 post-menopausal women with advanced breast cancer. Fourteen had responded to previous hormone therapy and 14 had no previous hormone therapy. There were two partial responses and two patients with stable disease for more than 6 months in the former group, and one partial response and two with stable disease for more than 6 months in the latter group. Toxicity was minimal. All responses occurred in soft tissue. Six out of seven patients who received tamoxifen after progression of disease on Zoladex showed a response. Peripheral oestradiol levels were measured, and they fell after 1 month from 33 pmol l-1 (+/- 20, s.d.) to 22 pmol l-1 (+/- 11, s.d.) (P less than 0.005). Responders and non-responders showed similar changes in oestradiol. Oestrone levels did not change significantly. These results suggest that Zoladex acts indirectly via changes in peripheral hormones, rather than directly on LHRH receptors on the tumour.


*Br. J. Cancer* (1989), **59**, 97–99

© The Macmillan Press Ltd., 1989

### Zoladex: Endocrine and therapeutic effects in post-menopausal breast cancer

A.L. Harris[1], J. Carmichael[1], B.M.J. Cantwell[1] & M. Dowsett[2]

[1]*University Department of Clinical Oncology, Regional Radiotherapy Centre, Newcastle General Hospital, Newcastle upon Tyne NE4 6BE and* [2]*Endocrine Department, Chelsea Hospital for Women, Dovehouse Street, London SW3 6LT, UK.*

**Summary** The endocrine and therapeutic effects of the LHRH agonist Zoladex have been assessed in 28 post-menopausal women with advanced breast cancer. Fourteen had responded to previous hormone therapy and 14 had no previous hormone therapy. There were two partial responses and two patients with stable disease for more than 6 months in the former group, and one partial response and two with stable disease for more than 6 months in the latter group. Toxicity was minimal. All responses occurred in soft tissue. Six out of seven patients who received tamoxifen after progression of disease on Zoladex showed a response. Peripheral oestradiol levels were measured, and they fell after 1 month from 33 $pmol\,l^{-1}$ ($\pm 20$, s.d.) to 22 $pmol\,l^{-1}$ ($\pm 11$, s.d.) ($P<0.005$). Responders and non-responders showed similar changes in oestradiol. Oestrone levels did not change significantly. These results suggest that Zoladex acts indirectly via changes in peripheral hormones, rather than directly on LHRH receptors on the tumour.

Recently luteinising hormone releasing hormone (LHRH) agonists have been shown to have direct inhibitory effects on human breast cancer cell lines *in vitro* and low affinity binding sites were demonstrated (Miller *et al.*, 1985; Blankenstein *et al.*, 1985). LHRH binding sites have also been demonstrated in primary breast tumours (Eidne *et al.*, 1985). Therefore, LHRH agonists such as Zoladex (Goserelin) have been assessed for potential efficacy in post-menopausal breast cancer, on the assumption that a therapeutic effect may indicate a direct antitumour effect (Plowman *et al.*, 1986; Waxman *et al.*, 1985), whereas in pre-menopausal women the major effect would be via ovarian suppression (Nicholson *et al.*, 1985). However, other endocrine effects may occur in post-menopausal women.

In the post-menopausal woman, adrenal and ovarian androgens are the main source of oestrogens (Judd *et al.*, 1982, 1974; Grodin *et al.*, 1973). They are converted peripherally by aromatase to oestrone and oestradiol. Inhibitors of aromatase (e.g. aminoglutethimide) are effective endocrine therapies in the treatment of post-menopausal breast cancer (Harris *et al.*, 1983*c*) and occasionally pre-menopausal breast cancer (Bezwoda *et al.*, 1987; Wander *et al.*, 1986). In the latter case, it has been suggested that intratumour conversion of androgens to oestrogens may be important and direct inhibition may be important, without any detectable lowering of peripheral oestrogens (Miller *et al.*, 1982; Bezwoda *et al.*, 1987).

Hydrocortisone alone can suppress adrenal androgen production (Harris *et al.*, 1984) and hence lower peripheral oestrogen levels (Harris *et al.*, 1984). We have recently shown that the androgens produced by the post-menopausal ovary are under pituitary FSH and LH control (Dowsett *et al.*, 1988). Zoladex may therefore have indirect endocrine effects on post-menopausal breast cancer. We have evaluated in this study the therapeutic effects of Zoladex and the correlation of response with peripheral endocrine changes in post-menopausal breast cancer patients.

### Materials and methods

Twenty-eight post-menopausal patients with locally advanced or progressive breast cancer were studied. Fourteen had no previous endocrine therapy and 14 had previously shown either stable disease for more than 6 months or partial response to other endocrine therapies.

Correspondence: A. L. Harris.
Received 8 July 1988; and in revised form, 13 August 1988.

Twelve patients had received Tamoxifen and three had a complete response, seven had a partial response and one stable disease. Eight had been given aminoglutethimide and four had a partial response. No patient had received chemotherapy. Other pre-treatment characteristics are shown in Table I.

Patients received monthly subcutaneous Zoladex, 3.6 mg, after infiltration of the local site with lignocaine. Response was assessed by UICC criteria (Hayward *et al.*, 1977). After Zoladex failure, patients who had received no previous endocrine therapy were treated with Tamoxifen 20 mg daily when appropriate.

Oestrone and oestradiol were measured by radio-immuno-assays which we have previously described (Harris *et al.*, 1983*a*; Dowsett *et al.*, 1987). These analyses have been specifically developed for the analysis of post-menopausal plasma oestrogen levels and have sensitivity limits of 30 and 3 $pmol\,l^{-1}$ respectively.

### Results

### *Responses to endocrine therapy*

Three patients showed partial responses for durations of 21 weeks and 141 weeks (continuing) and one patient died after 6 weeks from other causes. Four patients showed stable disease for more than 6 months (range 29–66 weeks). All responses were in soft tissues. Twenty-one patients had progressive disease.

Previous hormone therapy did not affect the likelihood of response to Zoladex. Of the 14 patients with previous endocrine therapy, two showed partial responses and two stable disease, while of the 14 with no previous endocrine treatment, one showed a partial response and two stable disease.

Toxicity was minimal, with no complaints of problems

**Table I** Pre-treatment characteristics

| | *Mean* | *Median* | *Range* |
|---|---|---|---|
| Age (years) | 67 | 70 | 32– 83 |
| Time from LMP (years) | 21 | 22 | 3– 39 |
| Disease-free interval (weeks) | 103 | 45 | 0–471 |
| Pre-treatment weight (kg) | 62 | 65 | 42–102 |
| Time from first relapse to start of Zoladex (weeks) | 73 | 2 | 0–508 |

Previous endocrine therapy, 14
Sites of disease: Soft tissue 23, lung 1, liver 1, nodes 9, bone 7.

with the injection sites. Five patients had mild nausea in the first few days after the initial Zoladex injection and one patient complained of increased post-menopausal flushes. Median survival from first recurrence was 305 weeks (log rank analysis). Median survival from start of Zoladex was 140 weeks (log rank analysis).

*Response to Tamoxifen after Zoladex*

Suitable patients who had not received prior hormone therapy were given Tamoxifen once disease progressed on Zoladex. Seven patients were thus treated and two with stable disease on Zoladex had a partial response to Tamoxifen. Of five with progressive disease on Zoladex, one had a complete response, two had a partial response (14 months and 7 months continuing) and one had stable disease (14 months continuing) on Tamoxifen.

**Figure 1** Effects of Zoladex on oestradiol and oestrone levels. Hormone levels were measured before therapy and 1 month later (*$P<0.005$). Bars represent s.e.m., $n=25$ paired observations. E1, oestrone; E2, oestradiol.

*Endocrine changes on Zoladex and response to therapy*

Oestradiol levels were significantly suppressed 1 month after starting therapy (paired $t$ test, $P<0.005$), but there was no significant depression of oestrone levels (Figure 1). Although average levels of oestrone fell, there was a wide variation overall.

The pre-treatment oestrone and oestradiol levels and post-treatment values did not differ between the responders (including those with stable disease) and non-responders (Table II). The patient with the best response did show the greatest suppression of oestradiol levels (43 to 13 pM, 71% reduction), which was maintained on therapy. Her oestrone levels were not suppressed.

Hormones were measured monthly in all patients. Oestradiol suppression was maintained and there was no evidence of hormone 'escape'.

### Discussion

This study shows that objective responses to an LHRH agonist can be obtained in post-menopausal breast cancer. Objective responses have been reported by Plowman *et al.* (1986) in two of ten previously untreated patients. Waxman *et al.* (1985) in a phase 2 study of another LHRH agonist found one minor response in 18 and that was in a previously untreated patient. In our study there were similar responses in the previously treated patients, although they were selected for previous response to other endocrine therapies.

Tamoxifen subsequently produced responses in six of seven patients in the group treated with Zoladex as initial therapy. These patients had favourable characteristics for response, i.e. soft tissue disease, few sites of disease and post-menopausal status. The low response rate to Zoladex is therefore unlikely to be due to intrinsic resistance to steroid hormone therapy, and the high response to Tamoxifen would be expected based on our selection criteria.

Other series report endocrine data as showing no significant changes in oestradiol or oestrone. However, details of the sensitivity of the assays were not presented in one study (Plowman *et al.*, 1986) and in the other the lower limit of detection of oestradiol was 50 pmol l$^{-1}$ (Waxman *et al.*, 1985). Clearly, this would fail to detect any significant change, since in our series the mean pre-treatment value was 33 pmol l$^{-1}$. Our series is larger than previously reported studies and small changes in oestradiol may not have been detected on lesser numbers of patients.

The study was carried out to test the hypothesis that LHRH agonists may have direct antitumour effects. The levels of Zoladex achieved *in vivo* are in the range 1–4 nM (Clayton *et al.*, 1985). Another LHRH agonist, Buserelin, inhibits the growth of the human breast cancer cell line MCF7 at concentrations of 1 nM or greater (Miller *et al.*, 1985; Foekens *et al.*, 1986). However, the affinity of the receptors is much lower than this – approximately 1 $\mu$M. Results with Zoladex *in vitro* have not been reported.

We have recently shown that the post-menopausal ovary is still stimulated by FSH and LH to produce androgens, and suppression of FSH and LH with Zoladex reduced androstenedione and testosterone plasma levels (Dowsett *et al.*, 1988). Since these steroids are precursors of oestrogens, the latter were also decreased. The suppression of oestradiol is more marked than that of oestrone and this may be due to the greater suppression of testosterone rather than androstenedione (Dowsett *et al.*, 1988), which are the respective precursors of the oestrogens.

Because of the peripheral endocrine effects, it is probable that the reductions in peripheral oestrogens are contributing to the therapeutic effect and the small reduction accounts for the low response rate compared to anti-oestrogens. It is interesting to note that the patient with the best response of longest duration showed the greatest fall in oestradiol levels. There was no general correlation of hormone suppression with response, and this has been noted for other hormone suppressive therapies (Santen *et al.*, 1982; Harris *et al.*, 1983*b*).

This study does not exclude LHRH receptors as a target for Zoladex, but we think that the suppression of oestradiol is a more likely explanation of the low response rate, whereas 20 of 30 patients reported by Eidne *et al.* (1985) had LHRH receptors in their tumours.

Because of its effect on suppression of ovarian androgens,

**Table II** Oestrone and oestradiol levels in responders and non-responders

| | | Oestrone(E1) | | | Oestradiol(E2) | | |
|---|---|---|---|---|---|---|---|
| | | Pre (pmol l$^{-1}$) | Post (pmol l$^{-1}$) | Mean % fall | Pre (pmol l$^{-1}$) | Post (pmol l$^{-1}$) | Mean % fall |
| Responders | Mean | 142 | 111 | 4 | 33 | 22 | 10 |
| | s.d. | 88 | 36 | 67 | 21 | 10 | 50 |
| Non-responders | Mean | 125 | 110 | 4 | 30 | 22 | 16 |
| | s.d. | 59 | 57 | 69 | 17 | 11 | 33 |

Zoladex should be further evaluated in combined endocrine therapy, since it may contribute to depletion of androgens that could be an intratumoral substrate for oestrogen production, as well as lowering peripheral oestradiol levels.

We thank Mr R. Wilson and Mr J. Farndon for referring patients for this study, Sister D. Simmons and M. Procter for blood sampling and Mrs V. Burnett for data collection. We thank ICI for support and drug supply.

### References

BEZWODA, W.R., MANSOOR, N. & DANSEY, R. (1987). Correlation of breast tumour aromatase activity and response to aromatase inhibition with aminoglutethimide. *Oncology*, **44**, 345.

BLANKENSTEIN, M.A., HENKELMAN, M.S. & KLIJN, J.G.M. (1985). Direct inhibitory effect of luteinizing hormone-releasing hormone agonist on MCF-7 human breast cancer cells. *Eur. J. Cancer Clin. Oncol.*, **21**, 1493.

CLAYTON, R.N., BAILEY, L.C., COTTAM, J., ARKELL, D., PERREN, T.J. & BLACKLEDGE, G.R.P. (1985). A radioimmunoassay for GnRH agonist analogue in serum of patients with prostate cancer treated with D-Ser (tBu)$^6$AZA Gly$^{10}$ GnRH. *Clin. Endocrinol.*, **22**, 453.

DOWSETT, M., CANTWELL, B.M.J.,, LAL, A., JEFFCOATE, S.L. & HARRIS, A.L. (1988). Suppression of postmenopausal ovarian steroidogenesis with the luteinizing hormone-releasing hormone antagonist goserelin. *J. Clin. Endocrinol. Metab.*, **66**, 672.

DOWSETT, M., GOSS, P.E., POWLES, T.J. & 4 others (1987). Use of the aromatase inhibitor 4-hydroxyandrostenedione in post-menopausal breast cancer: Optimization of therapeutic dose and route. *Cancer Res.*, **47**, 1957.

EIDNE, K.A., FLANAGAN, C.A. & MILLAR, R.P. (1985). Gonadotropin-releasing hormone binding sites in human breast carcinoma. *Science*, **229**, 989.

FOEKENS, J.A., HENKELMAN, M.S., FUKKINK, J.F., BLANKENSTEIN, M.A. & KLIJN, J.G.M. (1986). Combined effects of buserelin, estradiol and tamoxifen on the growth of MCF-7 human breast cancer cells *in vitro*. *Biochem. Biophys. Res. Commun.*, **140**, 550.

GRODIN, J.M., SIITERI, P.K. & MACDONALD, P.C. (1973). Source of estrogen production in postmenopausal women. *J. Clin. Endocrinol. Metab.*, **36**, 207.

HARRIS, A.L., DOWSETT, M., JEFFCOATE, S.L. & SMITH, I.E. (1983*a*). Aminoglutethimide dose and hormone suppression in advanced breast cancer. *Eur. J. Cancer Clin. Oncol.*, **19**, 493.

HARRIS, A.L., DOWSETT, M., SMITH, I.E. & JEFFCOATE, S. (1983*b*). Aminoglutethimide induced hormone suppression and response to therapy in advanced postmenopausal breast cancer. *Br. J. Cancer*, **48**, 585.

HARRIS, A.L., DOWSETT, M., SMITH, I.E. & JEFFCOATE, S. (1984). Hydrocortisone alone vs. hydrocortisone plus aminoglutethimide: A comparison of the endocrine effects in postmenopausal breast cancer. *Eur. J. Cancer Clin. Oncol.*, **20**, 463.

HARRIS, A.L., POWLES, T.J., SMITH, I.E. & 8 others (1983*c*). Aminoglutethimide for the treatment of advanced postmenopausal breast cancer. *Eur. J. Cancer Clin. Oncol.*, **19**, 11.

HAYWARD, J.L., CARBONE, P.P., HEUSON, J.C., KUMAOKA, S., SEGALOFF, A. & RUBENS, R.D. (1977). Assessment of response to therapy in advanced breast cancer. *Cancer*, **39**, 1284.

JUDD, H.L., JUDD, G.E., LUCAS, W.E. & YEN, S.S.C. (1974). Endocrine function of the postmenopausal ovary: Concentration of androgens and oestrogens in ovarian and peripheral vein blood. *J. Clin. Endocrinol. Metab.*, **39**, 1020.

JUDD, H.L., SHAMONKI, I.M., FRUMAR, A.M. & LAGASSE, L.D. (1982). Origin of oestradiol in postmenopausal women. *Obstet. Gynecol.*, **59**, 680.

MILLER, W.R., HAWKINS, R.A. & FORREST, A.P.M. (1982). Significance of aromatase activity in human breast cancer. *Cancer Res.*, **42**, Suppl., 3365.

MILLER, W.R., SCOTT, W.N., MORRIS, R., FRASER, H.M. & SHARPE, R.M. (1985). Growth of human breast cancer cells inhibited by a luteinizing hormone-releasing hormone agonist. *Nature*, **313**, 231.

NICHOLSON, R.I., WALKER, K.J., TURKES, A. & 4 others (1985). Endocrinological and clinical aspects of LHRH action (ICI 118630) in hormone dependent breast cancer. *J. Steroid Biochem.*, **23**, 843.

PLOWMAN, P.N., NICHOLSON, R.I. & WALKER, K.J. (1986). Remissions of post-menopausal breast cancer during treatment with the luteinising hormone releasing hormone agonist ICI 118630. *Br. J. Cancer*, **54**, 903.

RADFORD, J.A., KNIGHT, R.K. & RUBENS, R.D. (1985). Mitomycin C and vinblastine in the treatment of advanced breast cancer. *Eur. J. Cancer Clin. Oncol.*, **21**, 1475.

SANTEN, R.J., WORGUL, T.J., SAMOJLIK, E., BOUCHER, A.E., LIPTON, A. & HARVEY, H. (1982). Adequacy of estrogen suppression with aminoglutethimide and hydrocortisone as treatment of human breast cancer: Correlation of hormonal data with clinical responses. *Cancer Res.*, **42**, Suppl., 3397.

WANDER, H.E., BLOSSEY, H.CH. & NAGEL, G.A. (1986). Aminoglutethimide in the treatment of premenopausal patients with metastatic breast cancer. *Eur. J. Clin. Oncol.*, **22**, 1371.

WAXMAN, J.H., HARLAND, S.J., COOMBES, R.C. & 4 others (1985). The treatment of postmenopausal women with advanced breast cancer with buserelin. *Cancer Chemother. Pharmacol.*, **15**, 171.